# A supertree of Northern European macromoths

**DOI:** 10.1371/journal.pone.0264211

**Published:** 2022-02-18

**Authors:** Robert B. Davis, Erki Õunap, Toomas Tammaru

**Affiliations:** 1 Department of Zoology, Institute of Ecology and Earth Sciences, University of Tartu, Tartu, Estonia; 2 Institute of Agricultural and Environmental Sciences, Estonian University of Life Sciences, Tartu, Estonia; Nanjing Agricultural University, CHINA

## Abstract

Ecological and life-history data on the Northern European macromoth (Lepidoptera: Macroheterocera) fauna is widely available and ideal for use in answering phylogeny-based research questions: for example, in comparative biology. However, phylogenetic information for such studies lags behind. Here, as a synthesis of all currently available phylogenetic information on the group, we produce a supertree of 114 Northern European macromoth genera (in four superfamilies, with Geometroidea considered separately), providing the most complete phylogenetic picture of this fauna available to date. In doing so, we assess those parts of the phylogeny that are well resolved and those that are uncertain. Furthermore, we identify those genera for which phylogenetic information is currently too poor to include in such a supertree, or entirely absent, as targets for future work. As an aid to studies involving these genera, we provide information on their likely positions within the macromoth tree. With phylogenies playing an ever more important role in the field, this supertree should be useful in informing future ecological and evolutionary studies.

## Introduction

Ever more there is a push towards understanding how species are related to one another. While a small part of this is no doubt natural curiosity and the drive to reveal the Tree of Life in its entirety, the benefits of phylogenetic research for science are no way limited to such descriptive tasks. Besides the obvious use of phylogenetic information in taxonomy, there is increasing importance of using phylogenies as a tool in answering a wide array of evolutionary questions [1–3]. Different taxa are, however, unevenly covered by up-to-date phylogenetic information. The phylogenies of some groups of organisms–primarily vascular plants and vertebrate animals–have received much attention and are studied to an extent that allows various sophisticated phylogeny-based analyses to be performed [4, 5], though, generally speaking, such favourable situations are still rather an exception than the rule.

For insects in particular, phylogenetic coverage varies greatly among different taxa. There is little doubt that producing a phylogeny of all insect species would be a gargantuan task for various reasons; the size of such a phylogeny is one thing, but compared to many other groups, a large share of insect species is poorly known and, therefore, obtaining the necessary samples alone is a major issue. The problem with insects is also, to a large extent, a matter of geography. For example, we certainly have a more complete picture (phylogenetic or otherwise) of insect fauna at higher latitudes, such as for Europe and North America, than we do for the tropics [6]. As a final point on completeness, despite more and more discoveries, the insect fossil record remains patchy; for example, 35% of extant families have no record [7]. While our work here focuses on extant taxa, it is worth bearing in mind that a complete phylogeny would include extinct taxa too.

Within insects, butterflies and moths (Lepidoptera) have perhaps attracted most research attention. Our knowledge about the higher-level taxonomy of the order has recently considerably progressed as a consequence of large-scale, methodologically advanced molecular analyses [8, 9]; the picture at the (super)family level is clearing up. However, at a lower level, the picture is fragmentary. Butterflies (Papilionoidea) constitute a clade for which we are close to having a global phylogeny at the genus level [10], and a complete species-level phylogeny was recently published for the European fauna [11]. For the rest, there are major differences among taxonomic groups and regions in terms of the phylogenetic information available, with both amazingly detailed works and major gaps existing side by side. Indeed, research groups often focus on their own favourite taxa, which is entirely reasonable as research questions often demand such specialisation. The immediate demand in such cases–for example, to answer questions of comparative biology–is that phylogenies represent taxa for which data is available (e.g. on their ecology, anatomy, life history), and this is an important motivation in building a phylogeny. In this context, the lepidopteran fauna of Europe, especially of Northern Europe, is in an outstanding position. Due to the centuries-long, careful work of naturalists, we have substantial knowledge on the natural history of European moths, which is ready to be incorporated into phylogenetically based comparative studies [e.g. 12–14]. As things stand, the lack of sufficiently complete phylogenetic information has substantially hampered such developments.

The supertree approach involves summarising as much existing phylogenetic information as is feasible into one inclusive broad-scale phylogeny [15]. It provides one way to get as complete a picture as possible on the phylogeny of any group considering both lower and higher taxonomic levels; as outlined above, this is generally not the case for conventional phylogenetic studies. Just like any other phylogenetic tree, a supertree can be treated as a phylogenetic hypothesis and be used to answer research questions. Nevertheless, its value also lies in considering all available phylogenetic information up to a certain point in time and identifying those taxa for which current phylogenetic information is more robust and those for which relationships are problematic. Essentially, we can get an overview of the current state of play in this way. Therefore, compiling a supertree also allows us to assess just how well sampled a group is in general–just what proportion of taxa have even been considered? Consequently, points of focus for future phylogenetic work appear naturally, as those taxa with poor or no phylogenetic representation are easily identified.

In this paper, we present a supertree study of Northern European Macroheterocera (i.e. the lepidopteran clade embracing the superfamilies Drepanoidea, Geometroidea, Noctuoidea, Bombycoidea and Lasiocampoidea, see [16] for delimitation of this clade) at the genus level with two broad aims. Firstly, we provide the most inclusive phylogeny of the group to date, which is, in effect, a representation of over 30 years of research into algorithm-based macrolepidopteran phylogenetics. This will provide a phylogenetic framework for an ever-increasing number of ecological and evolutionary studies (likely of increasing scope and complexity) on taxa within the Northern European region for which data are readily available or easily obtained. Secondly, via the systematic stepwise approach taken, we summarise how sure we are about different regions of the phylogenetic tree–regions of the tree that we can be certain about, regions that we are uncertain about, and other regions that we have no information on at all. We are able to assess how well covered this group is in general, and where we should be focusing our efforts in the future to improve the picture.

## Materials and methods

### Taxonomy

**Fig 1:**
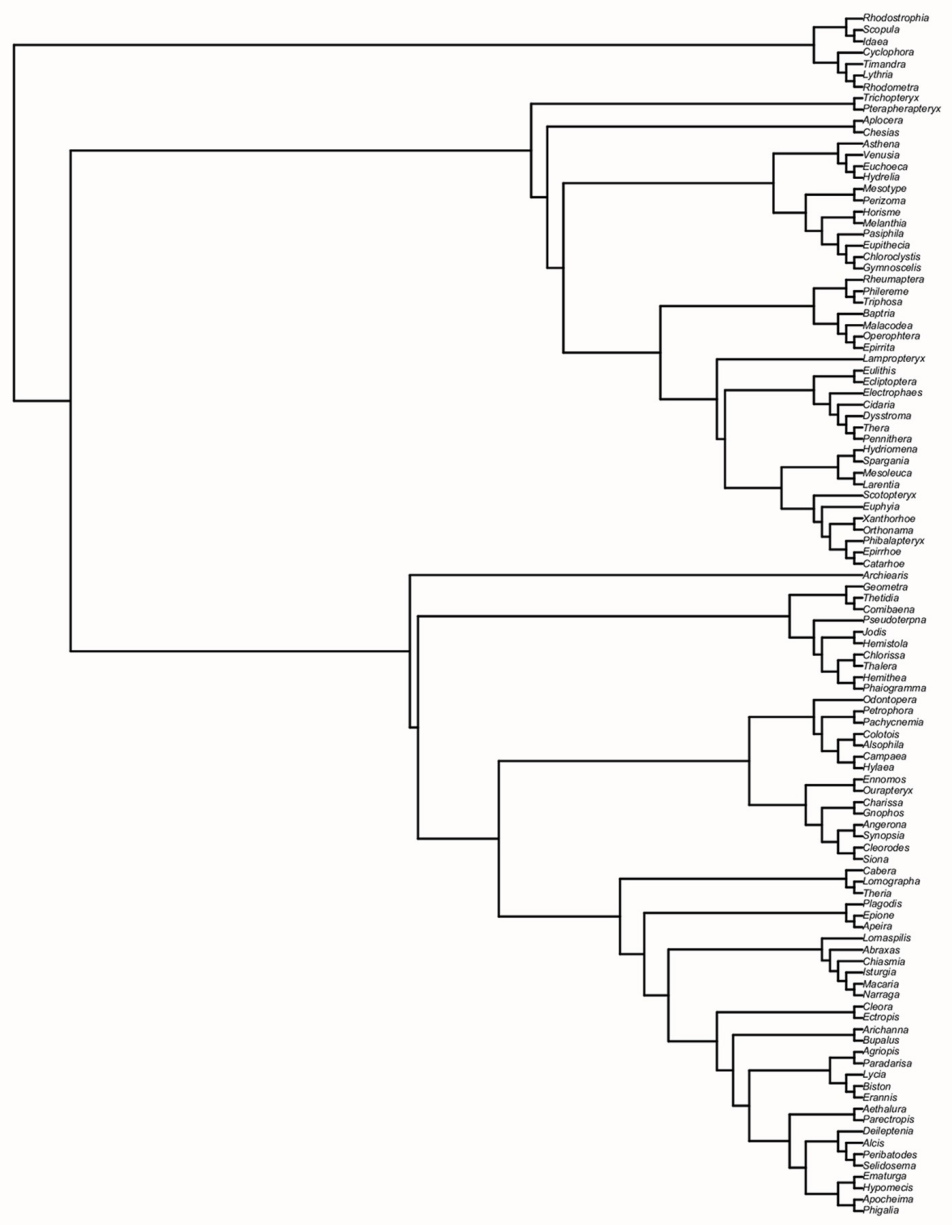
Phylogeny of Northern European Geometroidea based on the larger phylogeny of Murillo-Ramos et al. [21].

For consistency, the genus-level taxonomy of Aarvik et al. [17] was followed supplemented by that of Waring & Townsend [18]. Between them, these two references cover the entire region of Northern Europe, as described under the United Nations geoscheme (https://unstats.un.org/unsd/methodology/m49/). Only genera on the final list (S1 File) were considered for data collection. In the present supertree analysis, Geometroidea were, however, treated as a single taxon, with no internal structure of this superfamily analysed. We chose to do this for the following reasons. Firstly, the monophyly of Geometroidea, represented by families Geometridae and Uraniidae in Northern Europe, has not been contested in any recent major phylogenies [8, 9, 19]. Secondly, several comprehensive phylogenies of Geometridae subfamilies have recently been published [20–23]; as these trees include almost all Northern European genera of this family, a supertree approach is not needed for Geometridae. Thirdly, the authors of the present paper are close to completing work on a phylogenetic tree of Northern European Geometridae at the species level based on primary sequence data, which will provide more detail than any genus-level tree. However, it would be amiss to ignore the Geometroidea entirely, so we here present the tree of Murillo-Ramos et al. [21], pruned to show only Northern European genera. While this tree is not complete, it is still notably comprehensive, and we suggest using this as the most current phylogenetic hypothesis for the Northern European Geometroidea (Fig 1).

### Input tree collection and selection

Input trees were searched for via Web of Science using key words relating to phylogenetics (phylogen* clado* cladistic*–* denotes any word beginning with those letters), which follows Davis et al. [24], combined with taxonomy-related words concerning macromoths (see S2 File). Only studies focused on genera of interest were considered valid, and both the main texts of relevant studies and related supplementary material were searched for potential input trees. Studies focused on other groups, but, for example, using our target genera as outgroups, were not considered for input tree collection; these studies are less concerned about relationships of our target genera, even though technically they might provide some phylogenetic information on them. An additional point worth noting here is that various genera found in Northern Europe have representatives elsewhere. A study, for example, using North American species, but of genera also found in Northern Europe, would still provide relevant information for our purposes. Therefore, there was no attempt to filter by geographical region during the input tree search.

The field of phylogenetics is dynamic and new studies appear all the time, but supertree studies require a cut-off date for input tree collection. Our cut-off date was November 2020. At the other end of the time scale, studies were not considered if they were published prior to 1990, due to difficulty accessing earlier papers. We expect that the time window we conducted our input tree search in covers almost all relevant studies and very little extra information would be obtained by conducting the search in a larger window. Additionally, given time is a factor in conducting a supertree analysis, the amount of time required to search through earlier years is not justified. Not all potential input trees were readily available even within the 1990–Nov 2020 window, and we asked authors to provide papers which could conceivably have contained phylogenetic information, or for clearer figures where trees were unclear; however, not all authors responded, so some phylogenetic information from between these dates may be missing.

A final total of 191 potential input trees were collected from 124 studies (see S3 File). The word “potential” is used because a supertree analysis cannot be conducted without considering the issue of pseudoreplication (i.e. data non-independence). Here, pseudoreplication refers to the underlying data used to build these input trees and there is important discussion on this topic [e.g. 25, 26]. Protocols for removing (or in reality, reducing) such pseudoreplication exist, and we here follow a slightly modified version of that set out by Bininda-Emonds et al. [26]. These modifications are as follows: 1) because there is often at least some taxon overlap between input trees that use the same data type, we allow taxon overlap between two trees as long as it is 50% or less. As long at least half the taxa in each of two trees being compared are not found in the other tree, the two trees are retained in the data set. This allows inclusion of more phylogenetic information and better representation of taxa in the data set, whilst still eliminating the worst of the data non-independence; 2) A comprehensive tree is preferred to a more recent tree (these are alternative options in Bininda-Emonds et al. [26]) in cases where one input tree has to be chosen. This is to ensure maximum taxon representation in the data set; 3) Where it is not possible to choose between two or more trees (often the case within studies) we do not create a mini-supertree of these, but rather include all these input trees in the final analysis at a reduced weight–the weight is determined by just how many trees are involved (e.g. two trees get 50% weighting, four trees get 25% weighting). In doing this, we ensure the original phylogenies are included in the supertree analysis.

After dealing with the issue of pseudoreplication, taxa appearing in just one input tree were identified and removed from the data set. One input tree does not provide enough information on its own to place a taxon in a supertree; such singleton taxa were removed from the final analysis [27].

### Supertree analysis, matrix processing and tree support

The supertree was built using matrix representation with parsimony (MRP) [28, 29]. This is the most widely used supertree method and, despite some criticism [30, 31], has been shown to perform well in comparison with other methods both in simulation [32] and empirical studies [e.g. 24, 33]. In MRP, input trees are coded as a matrix of 0s and 1s, depending on the presence of a taxon either within (1) or outside (0) a clade. If a taxon is not present in an input tree, it receives a? in the matrix to denote that the data, in this case, is missing. Within the final matrix then, each node of each input tree is represented in the same way that a character would be in a matrix of morphological characters used in a cladistic analysis [28, 29, 34].

We used PAUP* [35] to run all analyses. We first attempted to analyse the final matrix as a whole using a heuristic parsimony search with TBR branch-swapping (other settings default, 1000 reps). However, a problem was encountered and the analysis failed to run past one repetition–the matrix contained far too many? s for a successful analysis. In situations where there are many? s in the matrix, which in our case seemed to be caused by low overlap between input trees (see discussion for more details), an MRP analysis may get stuck in the first repetition indefinitely and never complete. The minimum requirement is that an input tree should overlap with at least one other input tree by two taxa to be included in an MRP analysis unresolved [e.g. 15, 36], but in reality, as in our case, this may still not be enough overlap. In such situations, splitting the data set into smaller parts can relieve this issue [e.g. 33], which is the approach taken here. Our final data partitions were as follows: 1) Noctuidae; 2) Noctuoidea (with Noctuidae as a single taxon); 3) All non-noctuoid taxa, with Noctuoidea as a single taxon. These are clearly nested partitions, so it is important that the monophyly of these groups is uncontested; both the superfamily Noctuoidea and family Noctuidae are uncontroversial in this respect [e.g. 37].

Partitioning the data set enabled us to run the analysis successfully using the same software settings as outlined above. In some cases, some additional taxon removal was required (i.e. some taxa may appear in more than one input tree but are still represented by too few 0s and 1s in the matrix, which prevents the analysis running successfully–see discussion for more on this). The final data set combining the three partitions included 115 taxa (including Geometroidea as one), which is still far larger than any single input tree and makes this the largest genus-level study for Northern European macromoths to date. For each partition, more than one most parsimonious tree (MPT) was recovered, as expected (more details in Results), and both strict and 50% majority rule trees were generated.

Relying solely on the frequency with which a relationship is recovered in MPTs is not enough. Supertree support indices do exist, and we use the V index of Wilkinson et al. [38] to show how well supported relationships in the final supertree are by the input trees it is built from. Details can be found in Wilkinson et al. [38], but in summary, the V scale runs from -1 to +1, whereby -1 indicates that the input tree set offers no support for a relationship in the supertree and +1 indicates that the input tree set fully supports the relationship in the supertree. Values in between indicate differing degrees of conflict and support between input trees. We also refer to a less conservative version of the V index (V+), which considers input trees not directly conflicting with supertree relationships (e.g. polytomies) as support. Regarding a -1 support value, this would indicate a novel relationship not found in any input tree. This, of course, would be a point of concern and it can happen [39]. It is of the utmost importance that a supertree support metric such as V is used to assess the final supertree, not just to see which parts of the tree have the best support, but to assess the tree for such spurious relationships.

## Results and discussion

### The supertree

**Fig 2:**
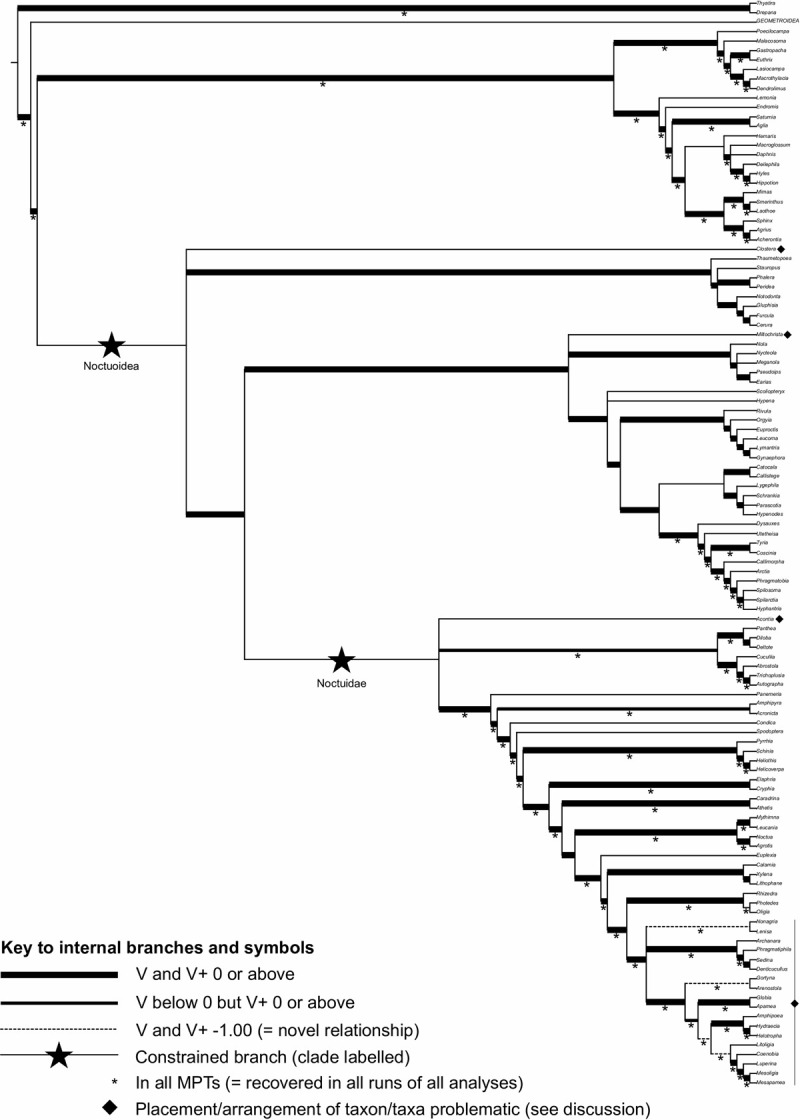
The majority rule genus-level supertree of North-European macromoths. Geometridae included at the family level (see main text and Fig 1). Branch thicknesses relate to V support. Dashed branches indicate novel, spurious relationships (V and V+ = -1). Large black stars on branches indicate clades that were constrained as monophyletic in order to partition the supertree analysis (Noctuoidea and Noctuidae); because these clades were constrained, they are not given a V score. Smaller stars below branches indicate those branches recovered in the strict consensus. Black diamond next to taxa indicate that their phylogenetic positions are discussed in the text.

The final supertree of Northern European macromoths comprised 115 taxa, including Geometroidea as a single taxon. Breaking down genus numbers by superfamily, excluding Geometroidea, superfamilies are represented as follows: Bombycoidea (16 genera), Drepanoidea (2 genera), Lasiocampoidea (7 genera), Noctuoidea (89 genera). The 50% majority rule consensus tree (Fig 2) is well resolved, though not completely and is mostly well supported by V scores (see S4 File for specific scores for clades). Overall, there are ten polytomies but all involve just three branches. Most of the polytomies appear in tipward positions and thus do not affect relationships among major taxa; three exceptions in deeper positions are discussed below. A considerable number of clades recovered were also found in the strict consensus tree, and these are also indicated on Fig 2 (S5 File).

The basic backbone of the supertree at the superfamily level is (Drepanoidea,(Geometroidea,(Noctuoidea,(Bombycoidea,Lasiocampoidea)))). A similar topology appears in all recent works on the high-level classification of Lepidoptera, though it seems that definitive consensus has not been reached with respect to whether Geometroidea or Noctuoidea constitute the sister clade of Bombycoidea + Lasiocampoidea [8, 9, 40, 41]. There is even a (Drepanoidea,(Bombycoidea,(Geometroidea,(Lasiocampoidea,Noctuoidea)))) arrangement of superfamilies recovered in the tree of Wahlberg et al. [19]. Notably, the number of unequivocal relationships is high in Bombycoidea and Lasiocampoidea for which major works on (or including) these clades [e.g. 40, 42–45] largely agree on higher-level classification. It is in the Noctuoidea, rather, where the three unresolved taxon placements alluded to above occur. In particular, curiously, the supertree provides no definitive support on the placement of *Clostera* within Noctuoidea, let alone within Notodontidae–something which appears not to have been questioned in any taxonomic treatment of the group [46]. The peculiar position of *Clostera* is most likely affected by the morphological studies of Miller [47, 48] and the early molecular study of Weller et al. [49], which placed this genus in a basal position relative to the rest of Noctuoidea. More recent, molecular works [37, 50] have consistently found *Clostera* to be a member of Notodontidae with high support, a conclusion which would not now likely be questioned. However, those earlier studies are independent lines of evidence and could not be discounted based on the methodological protocol employed in our study.

In the majority rule tree presented (Fig 2), Erebidae are well resolved, though, with the exception of the Arctiinae, fewer relationships are recovered in the strict consensus. The most unexpected result here is that Lithosiinae (represented here by *Miltochrista* only) is not recovered as sister to Arctiinae, as generally suggested, but sits as a part of a polytomy. There is a possible technical explanation for this; some Lithosiinae taxa were already removed prior to the final analysis simply because they were too poorly represented in the data set to be analysed. *Miltochrista*, while not being so poorly represented to prevent the analysis running, is still one taxon for which we have a relatively small amount of data, and it is possible that there is still too little phylogenetic information available on this taxon to place it confidently in the supertree. Arguably, *Miltochrista* could have also been removed from the analysis, but its inclusion is not detrimental to the rest of the supertree. Recent works [37, 51], however, unequivocally show a sister clade relationship between Arctiinae and Lithosiinae.

Overall, the sample of Noctuidae genera included in the present study shows a stable pattern of relationships. The polytomy at the very base of the clade reflects the controversy with respect to the higher classification of the family [52–54] so that the unresolved placement of *Acontia* is not surprising. Another somewhat problematic group is the tribe Apameini, represented in our study by a number of genera. Within Apameini, the problematic aspect is the occurrence of novel relationships not found in the input tree set. Such relationships should not be considered valid [e.g. 34] and we subscribe to this viewpoint. In our case, the cause of such a problem lies in taxon representation across input trees. In our final input tree set, of the taxa in this clade, only *Apamea* is found in more than two input trees, and all other taxa in this clade are in only the trees of Kergoat et al. [55] and Toussaint et al. [56]. In general, it can be expected that any conflict between just two input trees will not be easily resolved. Indeed, there is conflict between these two trees in the positions of the genera involved in these problematic supertree relationships. The chance of a supertree analysis generating unsupported relationships would likely be far lower with more sources of primary phylogenetic information to include in the final analysis.

From a practical perspective, and considering that the supertree is to be used as a phylogenetic hypothesis to aid future studies, we strongly suggest collapsing novel relationships into polytomies. What is crucial in the context of the supertree presented here is that there is good support for the more inclusive clades within which these unsupported relationships are found. For example, even if *Nonagria* + *Lenisa* is a spurious sister pairing, there is no argument that these taxa fall within a particular well-supported larger clade (from *Nonagria* to *Mesapamea* in Fig 2).

### Taxon representation

In order to get an overall view of how the supertree presented here is in terms of taxon representation it is worth considering how many Northern European taxa there are in total. These numbers are provided in Table 1.

**Table 1:** Northern European macromoth taxon numbers and supertree representation. As the supertree does not consider Geometroidea below superfamily level, numbers both including and excluding this superfamily are provided for family, genus and species levels, and alternative percentage coverages are given based on these accordingly; numbers in parentheses are when Geometroidea are excluded.

Taxonomic rank	Northern European number	Number of Northern European taxa represented in the supertree	% of Northern European taxa represented in supertree
Superfamily	5	5	100%
Family	12 (10)	10	83% (100%)
Genus	479 (325)	114	24% (35%)
Species	1096 (708)	312	28% (44%)

While complete at the superfamily and family levels, the final supertree (Fig 2) includes 115 taxa (114 genera + Geometroidea), which forms, however, just a subset of Northern European macromoth genera (N = 325 excluding Geometroidea, N = 479 including Geometroidea, see S1 File); this covers 35% of non-geometroid genera (Table 1). However, considering just those genera for which some phylogenetic information was available at the time the input tree search was conducted (N = 192 excluding Geometroidea), this proportion rises to 59% of genera. The genera in the supertree comprise 44% of Northern European non-geometroid macromoth species. As a side note on the Geometroidea, the tree from Murillo-Ramos et al. [21] presented in Fig 1 represents 106 of 154 (69%) Northern European geometroid genera, which comprise 84% of geometroid species in the region. Between the supertree and the geometroid tree of Murillo-Ramos et al. [21], 220 Northern European macromoth genera are represented phylogenetically.

Due to the requirements set by supertree-generating algorithms, we had to limit our final analyses to taxa that appeared in two or more input trees and were also well represented enough in the final data matrix (i.e. enough 0s and 1s in the matrix–see Materials and Methods). In addition to those 192 genera that have appeared in at least one published phylogeny, the number of genera for which some DNA sequence information is available (excluding those represented by DNA barcode sequences only) is 210 out of 325: Drepanoidea 11 (of 13), Bombycoidea 17 (of 17), Lasiocampoidea 9 (of 12) and Noctuoidea 173 (of 282) (S1 File). The coverage of the European moth fauna by data which have been, or can be used, in phylogenetic reconstruction is thus nowhere close to complete.

Moreover, the coverage remains markedly uneven across the subdivisions of Macroheterocera (S1 File). Curiously, for example, we are unaware of any phylogenetic treatment of the family Drepanidae, though some species have appeared in a few works, considered as input for the present study [e.g. 40, 57, 58]. Due to recent efforts [e.g. 20–23], the coverage of Geometridae by phylogenetic studies has been increasing rapidly; representatives of at least 127 Northern European genera (out of 153) have been included in molecular phylogenies. The situation is also relatively favourable for Erebidae, largely again thanks to a few major studies [59–61]. For Noctuidae, the coverage is much poorer, with just 56% of genera with some phylogenetic information available. The gaps are primarily found in the largest subfamily Noctuinae, in which some large tribes like Xylenini, Hadenini and Orthosiini have so far received little attention in phylogenetic studies.

### Implications

We see the primary use of the supertree derived in the present paper, as for other supertrees, in providing phylogenetic information for cross-species studies in evolutionary ecology [e.g. 62–64] and potentially in a wide array of other fields within comparative biology [34, 65], or for instance in ecological studies using phylogenetically weighted diversity indices [66, 67]. The tree can be used for planning research, both when designing how to sample study species across the phylogeny, and when evaluating the availability of phylogenetic information necessary for the research planned.

Although as comprehensive as currently possible, the presented supertree (Fig 2) contains only a fraction of genera for which phylogenetic information is available (S1 File). This is a consequence of specific limitations associated with the application of the supertree method, discussed above. The presented supertree can nevertheless be used as a basis for generating more comprehensive trees incorporating such species with phylogenetic information available. To facilitate such actions, we have produced a table (Table 2) indicating where in the supertree additional genera might fit; this is based on the positions of those genera removed from input trees prior to supertree analysis. Should such genera need including in a phylogeny in addition to those already in the supertree, their placement can be estimated based on this information.

**Table 2:** Genera removed from analysis with probable positions in the phylogeny based on published phylogenetic studies.

Genus (or groups of genera)	Taxonomic position (Aarvik et al. [17])	Closest genus/genera in supertree	Reference(s)
*Habrosyne*, *Tethea*	Drepanidae: Thyatirinae	Sister genera. Together sister to *Drepana*	[40]
*Cilix*	Drepanidae: Drepaninae	*Drepana*	[57]
*Odonestis*	Lasiocampidae: Lasiocampinae	*Euthrix* or *Gastropacha*	[44]
*Proserpinus*	Sphingidae: Macroglossinae	Different positions within Macroglossinae	[68]
*Pheosia*, *Odontosia*	Notodontidae: Notodontinae (*Pheosia*), Ptilodontinae (*Odontosia*)	Miller [47] has these as sister taxa, together sister to *Notodonta*. Kawahara et al. [8] puts *Pheosia* at base of Noctuoidea	[8, 47]
*Ptilophora*	Notodontidae: Ptilodontinae	Different positions in a clade containing *Furcula*, *Gluphisia*, and *Cerura*	[47, 53]
*Harpyia*	Notodontidae: Dicranurinae	*Stauropus*	[47]
*Colobochyla*	Erebidae: Hypeninae	*Hypena*	[37, 59]
*Arctornis*	Erebidae: Lymantriinae: Arctorthnithini	Different positions within Lymantriinae	[37, 59, 69]
*Calliteara*, *Laelia*, *Sphrageidus*	Erebidae: Lymantriinae: Orgyiini (*Calliteara*, *Laelia*), Nygmiini (*Sphrageidus*)	Either appear in one or both of these refs. Close to *Orgyia* in Wang et al. [69]. Of these taxa, only *Calliteara* in Zhang et al. [70]: placed in Erebidae	[69, 70]
*Callopistria*	Noctuidae: Eriopinae	*Agrotis*	[70]
*Apantesis*, *Chelis*, *Diacrisia*	Erebidae: Arctiinae: Arctiini	Rönkä et al. [61] places them as sister to *Arctia*, *Chelis* as sister to this clade, and *Diacrisia* as sister to this. Zaspel et al. [71] has *Apantesis* as part of a paraphyletic assembledge of Arctiinae close to *Arctia*	[61, 71]
*Atolmis*, *Cybosia*, *Eilema*, *Lithosia*, *Pelosia*, *Setina*	Erebidae: Arctiinae: Lithosiini	Either appear in one or both of these refs. Dowdy et al. [72] has *Atolmis* as sister to *Miltochrista* and Scott et al. [74] have three of them in a clade as sister to *Miltochrista*, Wink & Von Nickisch-Rosenegk [76] have *Atolmis* and *Eilema* as sisters within Arctiinae. Scott & Branham [75] put *Pelosia* in an undetermined position with *Lithosia* and *Eilema* and put *Setina* in an undetermined position associated with all these taxa. Zenker et al. [73] have *Setina* as sister to *Miltochrista*	[72–76]
*Diaphora*	Erebidae: Arctiinae: Arctiini	*Hyphantria* or *Spilosoma*	[61, 76]
*Euplagia*	Erebidae: Arctiinae: Arctiini	*Callimorpha*	[61, 71, 73]
*Calyptra*	Erebidae: Calpinae	Within Erebidae at the base of a clade containing Erebinae, Boletobiinae, Toxocampinae	[37, 59]
*Herminia*, *Paracolax*, *Polypogon*, *Simplicia*	Erebidae: Herminiinae	Together as one clade, which is sister to Arctiinae	[37, 59]
*Pechipogo*	Erebidae: Herminiinae	Arctiinae	[57]
*Eublemma*, *Laspeyria*, *Phytometra*, *Trisateles*	Erebidae: Boletobiinae	Either appear in one or both of these refs and make up one clade. *Trisateles* sister to *Parascotia* and the whole clade sister to one containing *Hypenodes* and *Schrankia* (polytomy here in supertree)	[37, 59]
*Catephia*, *Euclidia*	Erebidae: Erebinae	*Euclidia* sister to *Callistege*, *Catephia* base of a clade containing these taxa and *Catocala*	[37,59]
*Dysgonia*, *Grammodes*	Erebidae: Erebinae	*Grammodes*, when it appears, is placed as sister to *Catocala*, except where *Dysgonia* is also included (Sun et al. [79]); then *Dysgonia* and *Grammodes* together is the sister group to *Catocala*. Homziak et al. [80] place *Dysgonia* as sister to *Callistege*, and these two together as sister to *Catocala*	[77–84]
*Moma*	Noctuidae: Dyopsinae	Rota et al. [86] place is as sister to *Acontia*. Keegan et al. [85] has it at the base of Noctuidae	[85, 86]
*Chrysodeixis*, *Ctenoplusia*, *Diachrysia*, *Macdunnoughia*, *Plusia*, *Syngrapha*, *Thysanoplusia*	Noctuidae: Plusiinae	Nomura [107] groups these taxa together. Only *Ctenoplusia* appears in the other references where it is in different positions within Noctuidae	[50, 77–79, 81–84, 87–107]
*Colocasia*	Noctuidae: Pantheinae	*Panthea*	[108]
*Trichosea*	Noctuidae: Pantheinae	*Cucullia*	[40, 53]
*Brachionycha*	Noctuidae: Amphipyrinae: Psaphidini	*Amphipyra*	[37, 57, 85, 86, 109]
*Craniophora*	Noctuidae: Acronictinae	*Acronicta*	[37, 57, 85, 86, 109]
*Sympistis*	Noctuidae: Oncocnemidinae	*Panemeria*	[85]
*Aedia*	Noctuidae: Aediinae	*Condica*	[85, 86]
*Actinotia*, *Hoplodrina*	Noctuidae: Noctuinae: Actinotiini (*Actinotia*), Caradrinini (*Hoplodrina*)	In a clade containing *Noctua* and Apamea, with various arrangements of taxa	[37, 57, 85, 86, 109]
*Anarta*	Noctuidae: Noctuinae: Hadenini	*Mythimna*	[77, 78]
*Aporophyla*	Noctuidae: Noctuinae: Xylenini	Within Noctuinae, on its own branch along the backbone of the tree	[56]
*Celaena*	Noctuidae: Noctuinae: Apameini	*Litoglia*	[56]
*Conistra*	Noctuidae: Noctuinae: Xylenini	*Xylena*	[108]
*Crypsedra*	Noctuidae: Noctuinae: Apameini	*Calamia*	[55]
*Diarsia*, *Euxoa*, *Ochropleura*, *Peridroma*, *Spaelotis*, *Xestia*	Noctuidae: Noctuinae: Noctuini	All these taxa are together in a clade with *Euxoa* sister to *Agrotis*	[108]
*Eremobia*	Noctuidae: Noctuinae: Apameini	*Arenostola*	[55]
*Hyppa*	Noctuidae: Noctuinae: Xylenini	Different positions in a clade containing *Apamea*, *Oligia*, *Lithophane*, *Xylena* (and *Conistra*)	[108]
*Lasionycta*, *Polia*	Noctuidae: Noctuinae: Eriopygini (*Lasionycta*), Hadenini (*Polia*)	Sister taxa, basal to a clade containing *Euplexia* (and *Phlogophora*), *Apamea*, *Oligia*, *Lithophane*, *Xylena* (and *Conistra* and *Hyppa*)	[108]
*Phlogophora*	Noctuidae: Noctuinae: Phlogophorini	*Euplexia*	[108]
*Mamestra*	Noctuidae: Noctuinae: Hadenini	*Mythimna*	[81, 83]
*Oria*	Noctuidae: Noctuinae: Apameini	Sister to a clade containing *Amphipoea*, *Helotropha* and *Hydraecia*	[55]
*Orthosia*	Noctuidae: Noctuinae: Orthosiini	*Mythimna*	[108]
*Pseudeustrotia*	Noctuidae: Noctuinae: Pseudeustrotiini	*Condica*	[53]
*Staurophora*	Noctuidae: Noctuinae: Apameini	*Calamia*	[56]
*Tholera*	Noctuidae: Noctuinae: Tholerini	Within Noctuinae, on its own branch along the backbone of the tree	[56]

The manual insertion of extra genus- and species-level phylogenetic information into the supertree should be technically straightforward, especially given that the supertree does not have branch lengths to consider. Certainly for many methods that incorporate phylogenetic information, branch lengths are important (e.g. various phylogenetic comparative methods, ancestral state reconstruction methods, phylogenetic distance methods). Just as for any other phylogeny lacking branch lengths, the supertree can be dated as required to provide these [e.g. 64]. Regardless, in the context of this paper, branch lengths are not crucial for assessing taxonomic coverage and branching patterns.

The other key use of the supertree is to indicate the current state of phylogenetic knowledge about the lepidopteran fauna of (Northern) Europe. We discussed above how uneven current coverage is, and if a more even coverage of the moth fauna is desired, further studies might prioritize certain taxa–for example Drepanidae and the noctuid tribe Xylenini–to facilitate comparative studies incorporating the ecological diversity of European moth fauna to the largest possible extent.

### Methodological aspects

Supertrees are designed to be comprehensive in their taxonomic coverage, but they can only be constructed based on phylogenetic information already available [e.g. 34]. From a purely practical point of view, it is desired that input trees overlap in their taxonomic composition to a large extent, and that the underlying data is different between input trees to prevent issues with data non-independence. In general, higher taxonomic overlap [e.g. 32], and a lower degree of data-type overlap [e.g. 25] leads to a better supertree analysis. These two points are considered here in turn in the context of the Northern European Macrolepidoptera supertree.

The extent of taxonomic overlap required between input trees is something that has been widely discussed. At the very least, an input tree should share at least two taxa with one other input tree otherwise the supertree will be entirely unresolved [15, 36]. However, as seen via the effort to generate a supertree in this study, this minimum requirement does not guarantee that a data set can be successfully analysed.

The proportion of taxa that input trees share is one aspect of taxonomic overlap–one of taxon numbers–but another challenge related to taxonomic overlap is focused on specific taxa themselves. The more input trees a taxon appears in, the more information it will be represented by in the matrix to be analysed–quite obviously, fewer gaps in the matrix are better (see Materials & Methods). Within our input tree set, there are examples of certain taxa appearing in a just a limited subset of input trees, for example, as seen with many Apameini genera. This kind of limitation can lead to analytical problems, especially where conflict over a taxon’s position is great between these few input trees. Beyond the taxa that could be included in our final analysis, even with the challenges they brought, there are those taxa appearing in just one input tree; with no overlap with another input tree, this means they cannot be considered for inclusion in the supertree analysis [e.g. 27]. With the Northern European Macrolepidoptera in mind, accessing samples of these genera should not be a problem and should be considered in future phylogenetic analyses.

On the subject of the problem of data non-independence–that is, how much primary molecular or morphological data is replicated across input trees–the message is clear; this has to be minimised in supertree studies [25, 26, 31, 110]. As seen from the Northern European Macrolepidoptera data set, data non-independence is a genuine issue and can be difficult to resolve; for example, there are several potential input trees based on the 13 protein-coding genes of the mitochondrial genome and they tend to use partially overlapping set of taxa (S3 File). Including all such studies would lead to a disproportionally large contribution of these specific genome data to supertree relationships and out of all potential input trees, the most comprehensive trees are used (e.g. for example, Seo et al. [81], Wu et al. [82] for these 13 mitochondrial protein-coding genes). In particular reference to molecular data, it is probably unrealistic to expect that researchers will avoid using the same genes or combinations of them in the future. While taxonomic overlap is something that will improve over time, data non-independence is an issue that supertree research will have to continue to face.

## Conclusions

Here we have presented a supertree of Northern European macromoth genera, which is the most comprehensive phylogeny of the group to date (114 genera + Geometroidea). The intent is to show our current state of knowledge on the phylogeny of this group, where we can be almost certain of relationships and where uncertainty currently lies. This tree should serve as a basis and as motivation for researchers who intend to resolve the phylogeny of these macromoths by providing target areas of the tree for further investigation (e.g. where polytomies exist) and in identifying target taxa for which phylogenetic information is currently either sparse or non-existent. The presented supertree, in combination with the recently published trees on the superfamily Geometroidea, is nevertheless useable as a phylogenetic hypothesis in its current state, as input information for phylogenetic comparative analyses, for example, and also as an aid for those planning comparative analyses, indicating those groups of Lepidoptera in which sufficient amount of phylogenetic information is available.

## Supporting information

S1 File Valid genus list with information on phylogeny and molecular data availability. (XLSX)

S2 File Input tree search terms for the Macrolepidoptera supertree. (XLSX)

S3 File Input tree list. (XLSX)

S4 File Supertree nodal support (V index scores). (DOCX)

S5 File Strict-consensus supertree. (TIF)
