# Supplementary material for: A supertree of Northern European macromoths
Source: PLoS One. 2022 Feb 18;17(2):e0264211. doi: 10.1371/journal.pone.0264211 (PMC8856531; doi:10.1371/journal.pone.0264211)
Supplement: S4 File — (DOCX) [file pone.0264211.s004.docx]

**SUPPLEMENTARY FILE S4**

Supertree nodal support (V index scores)

These are the V index scores for clade groupings based on the method of Wilkinson et al. (2005). Each clade in the supertree is represented along with its V score (v1) and V+ (v2, less conservative) variant. Also, provided is information on how many input trees support (S), conflict with (Q) or permit (P) the supertree clade. See Wilkinson et al (2005) for more details.

(Lemonia,Endromis,Saturnia,Aglia,Hemaris,Macroglossum,Hyles,Hippotion,Deilephila,Daphnis,Mimas,Smerinthus,Laothoe,Sphinx,Agrius,Acherontia) S=288 Q=12 P=24 v1=0.92 v2=0.925926

(Endromis,Saturnia,Aglia,Hemaris,Macroglossum,Hyles,Hippotion,Deilephila,Daphnis,Mimas,Smerinthus,Laothoe,Sphinx,Agrius,Acherontia) S=264 Q=36 P=24 v1=0.76 v2=0.777778

(NOCTUOIDEA,Poecilocampa,Malacosoma,Gastropacha,Euthrix,Lasiocampa,Macrothylacia,Dendrolimus,Lemonia,Endromis,Saturnia,Aglia,Hemaris,Macroglossum,Hyles,Hippotion,Deilephila,Daphnis,Mimas,Smerinthus,Laothoe,Sphinx,Agrius,Acherontia) S=276 Q=84 P=0 v1=0.533333 v2=0.533333

(Poecilocampa,Malacosoma,Gastropacha,Euthrix,Lasiocampa,Macrothylacia,Dendrolimus,Lemonia,Endromis,Saturnia,Aglia,Hemaris,Macroglossum,Hyles,Hippotion,Deilephila,Daphnis,Mimas,Smerinthus,Laothoe,Sphinx,Agrius,Acherontia) S=324 Q=12 P=24 v1=0.928571 v2=0.933333

(Thyatira,Drepana,GEOMETROIDEA,NOCTUOIDEA,Poecilocampa,Malacosoma,Gastropacha,Euthrix,Lasiocampa,Macrothylacia,Dendrolimus,Lemonia,Endromis,Saturnia,Aglia,Hemaris,Macroglossum,Hyles,Hippotion,Deilephila,Daphnis,Mimas,Smerinthus,Laothoe,Sphinx,Agrius,Acherontia) S=360 Q=0 P=0 v1=1 v2=1

(GEOMETROIDEA,NOCTUOIDEA,Poecilocampa,Malacosoma,Gastropacha,Euthrix,Lasiocampa,Macrothylacia,Dendrolimus,Lemonia,Endromis,Saturnia,Aglia,Hemaris,Macroglossum,Hyles,Hippotion,Deilephila,Daphnis,Mimas,Smerinthus,Laothoe,Sphinx,Agrius,Acherontia) S=330 Q=30 P=0 v1=0.833333 v2=0.833333

(Saturnia,Aglia,Hemaris,Macroglossum,Hyles,Hippotion,Deilephila,Daphnis,Mimas,Smerinthus,Laothoe,Sphinx,Agrius,Acherontia) S=284 Q=16 P=24 v1=0.893333 v2=0.901235

(Hemaris,Macroglossum,Hyles,Hippotion,Deilephila,Daphnis,Mimas,Smerinthus,Laothoe,Sphinx,Agrius,Acherontia) S=240 Q=0 P=84 v1=1 v2=1

(Mimas,Smerinthus,Laothoe,Sphinx,Agrius,Acherontia) S=144 Q=24 P=84 v1=0.714286 v2=0.809524

(Sphinx,Agrius,Acherontia) S=104 Q=0 P=148 v1=1 v2=1

(Agrius,Acherontia) S=24 Q=0 P=108 v1=1 v2=1

(Mimas,Smerinthus,Laothoe) S=48 Q=12 P=84 v1=0.6 v2=0.833333

(Smerinthus,Laothoe) S=48 Q=0 P=72 v1=1 v2=1

(Hemaris,Macroglossum,Hyles,Hippotion,Deilephila,Daphnis) S=136 Q=8 P=132 v1=0.888889 v2=0.942029

(Macroglossum,Hyles,Hippotion,Deilephila,Daphnis) S=80 Q=0 P=184 v1=1 v2=1

(Hyles,Hippotion,Deilephila) S=60 Q=0 P=120 v1=1 v2=1

(Hyles,Hippotion) S=24 Q=12 P=144 v1=0.333333 v2=0.866667

(Saturnia,Aglia) S=36 Q=0 P=156 v1=1 v2=1

(Poecilocampa,Malacosoma,Gastropacha,Euthrix,Lasiocampa,Macrothylacia,Dendrolimus) S=120 Q=0 P=60 v1=1 v2=1

(Malacosoma,Gastropacha,Euthrix,Lasiocampa,Macrothylacia,Dendrolimus) S=108 Q=0 P=72 v1=1 v2=1

(Gastropacha,Euthrix,Lasiocampa,Macrothylacia,Dendrolimus) S=108 Q=0 P=48 v1=1 v2=1

(Lasiocampa,Macrothylacia,Dendrolimus) S=42 Q=3 P=99 v1=0.866667 v2=0.958333

(Macrothylacia,Dendrolimus) S=12 Q=0 P=108 v1=1 v2=1

(Gastropacha,Euthrix) S=9 Q=0 P=75 v1=1 v2=1

(Thyatira,Drepana) S=24 Q=0 P=60 v1=1 v2=1

(Clostera,Thaumetopoea,Stauropus,Phalera,Peridea,Notodonta,Gluphisia,Furcula,Cerura,NOCTUIDAE,Miltochrista,Nola,Nycteola,Meganola,Pseudoips,Earias,Scoliopteryx,Hypena,Rivula,Orgyia,Leucoma,Lymantria,Gynaephora,Euproctis,Lygephila,Schrankia,Parascotia,Hypenodes,Catocala,Callistege,Dysauxes,Utetheisa,Tyria,Coscinia,Callimorpha,Phragmatobia,Spilosoma,Spilarctia,Hyphantria,Arctia) S=162 Q=0 P=6 v1=1 v2=1

(NOCTUIDAE,Miltochrista,Nola,Nycteola,Meganola,Pseudoips,Earias,Scoliopteryx,Hypena,Rivula,Orgyia,Leucoma,Lymantria,Gynaephora,Euproctis,Lygephila,Schrankia,Parascotia,Hypenodes,Catocala,Callistege,Dysauxes,Utetheisa,Tyria,Coscinia,Callimorpha,Phragmatobia,Spilosoma,Spilarctia,Hyphantria,Arctia) S=138 Q=6 P=12 v1=0.916667 v2=0.923077

(Miltochrista,Nola,Nycteola,Meganola,Pseudoips,Earias,Scoliopteryx,Hypena,Rivula,Orgyia,Leucoma,Lymantria,Gynaephora,Euproctis,Lygephila,Schrankia,Parascotia,Hypenodes,Catocala,Callistege,Dysauxes,Utetheisa,Tyria,Coscinia,Callimorpha,Phragmatobia,Spilosoma,Spilarctia,Hyphantria,Arctia) S=96 Q=39 P=21 v1=0.422222 v2=0.5

(Scoliopteryx,Hypena,Rivula,Orgyia,Leucoma,Lymantria,Gynaephora,Euproctis,Lygephila,Schrankia,Parascotia,Hypenodes,Catocala,Callistege,Dysauxes,Utetheisa,Tyria,Coscinia,Callimorpha,Phragmatobia,Spilosoma,Spilarctia,Hyphantria,Arctia) S=123 Q=12 P=15 v1=0.822222 v2=0.84

(Rivula,Orgyia,Leucoma,Lymantria,Gynaephora,Euproctis,Lygephila,Schrankia,Parascotia,Hypenodes,Catocala,Callistege,Dysauxes,Utetheisa,Tyria,Coscinia,Callimorpha,Phragmatobia,Spilosoma,Spilarctia,Hyphantria,Arctia) S=96 Q=27 P=27 v1=0.560976 v2=0.64

(Lygephila,Schrankia,Parascotia,Hypenodes,Catocala,Callistege,Dysauxes,Utetheisa,Tyria,Coscinia,Callimorpha,Phragmatobia,Spilosoma,Spilarctia,Hyphantria,ArctiaS=70 Q=26 P=36 v1=0.458333 v2=0.606061

(Dysauxes,Utetheisa,Tyria,Coscinia,Callimorpha,Phragmatobia,Spilosoma,Spilarctia,Hyphantria,Arctia) S=69 Q=9 P=30 v1=0.769231 v2=0.833333

(Utetheisa,Tyria,Coscinia,Callimorpha,Phragmatobia,Spilosoma,Spilarctia,Hyphantria,Arctia) S=69 Q=9 P=30 v1=0.769231 v2=0.833333

(Tyria,Coscinia,Callimorpha,Phragmatobia,Spilosoma,Spilarctia,Hyphantria,ArctiaS=63 Q=0 P=45 v1=1 v2=1

(Callimorpha,Phragmatobia,Spilosoma,Spilarctia,Hyphantria,Arctia) S=66 Q=0 P=42 v1=1 v2=1

(Phragmatobia,Spilosoma,Spilarctia,Hyphantria,Arctia) S=54 Q=0 P=54 v1=1 v2=1

(Phragmatobia,Spilosoma,Spilarctia,Hyphantria) S=36 Q=0 P=60 v1=1 v2=1

(Spilosoma,Spilarctia,Hyphantria) S=36 Q=0 P=54 v1=1 v2=1

(Tyria,Coscinia) S=12 Q=0 P=18 v1=1 v2=1

(Lygephila,Schrankia,Parascotia,Hypenodes,Catocala,Callistege) S=18 Q=0 P=66 v1=1 v2=1

(Catocala,Callistege) S=18 Q=0 P=66 v1=1 v2=1

(Lygephila,Schrankia,Parascotia,Hypenodes) S=12 Q=0 P=0 v1=1 v2=1

(Schrankia,Parascotia,Hypenodes) S=12 Q=0 P=0 v1=1 v2=1

(Rivula,Orgyia,Leucoma,Lymantria,Gynaephora,Euproctis) S=48 Q=12 P=54 v1=0.6 v2=0.789474

(Orgyia,Leucoma,Lymantria,Gynaephora,Euproctis) S=60 Q=0 P=54 v1=1 v2=1

(Leucoma,Lymantria,Gynaephora,Euproctis) S=54 Q=0 P=36 v1=1 v2=1

(Leucoma,Lymantria,Gynaephora) S=36 Q=0 P=48 v1=1 v2=1

(Lymantria,Gynaephora) S=24 Q=0 P=60 v1=1 v2=1

(Nola,Nycteola,Meganola,Pseudoips,Earias) S=24 Q=0 P=36 v1=1 v2=1

(Nycteola,Meganola,Pseudoips,Earias) S=24 Q=0 P=36 v1=1 v2=1

(Pseudoips,Earias) S=12 Q=0 P=18 v1=1 v2=1

(Thaumetopoea,Stauropus,Phalera,Peridea,Notodonta,Gluphisia,Furcula,Cerura) S=40 Q=14 P=42 v1=0.481481 v2=0.708333

(Stauropus,Phalera,Peridea,Notodonta,Gluphisia,Furcula,Cerura) S=40 Q=8 P=48 v1=0.666667 v2=0.833333

(Notodonta,Gluphisia,Furcula,Cerura) S=18 Q=0 P=48 v1=1 v2=1

(Gluphisia,Furcula,Cerura) S=18 Q=0 P=36 v1=1 v2=1

(Furcula,Cerura) S=12 Q=0 P=24 v1=1 v2=1

(Phalera,Peridea) S=6 Q=0 P=66 v1=1 v2=1

(Panthea,Diloba,Deltote,Cucullia,Trichoplusia,Autographa,Abrostola) S=18 Q=24 P=18 v1=-0.142857 v2=0.2

(Panthea,Diloba,Deltote) S=12 Q=0 P=24 v1=1 v2=1

(Spodoptera,Pyrrhia,Schinia,Heliothis,Helicoverpa,Elaphria,Cryphia,Caradrina,Athetis,Euplexia,Xylena,Lithophane,Calamia,Rhizedra,Photedes,Oligia,Nonagria,Lenisa,Phragmatiphila,Sedina,Denticucullus,Archanara,Gortyna,Arenostola,Globia,Apamea,Litoligia,Mesoligia,Mesapamea,Luperina,Coenobia,Hydraecia,Helotropha,Amphipoea,Mythimna,Leucania,Noctua,Agrotis) S=84 Q=40 P=2 v1=0.354839 v2=0.365079

(Pyrrhia,Schinia,Heliothis,Helicoverpa,Elaphria,Cryphia,Caradrina,Athetis,Euplexia,Xylena,Lithophane,Calamia,Rhizedra,Photedes,Oligia,Nonagria,Lenisa,Phragmatiphila,Sedina,Denticucullus,Archanara,Gortyna,Arenostola,Globia,Apamea,Litoligia,Mesoligia,Mesapamea,Luperina,Coenobia,Hydraecia,Helotropha,Amphipoea,Mythimna,Leucania,Noctua,Agrotis) S=84 Q=42 P=0 v1=0.333333 v2=0.333333

(Elaphria,Cryphia,Caradrina,Athetis,Euplexia,Xylena,Lithophane,Calamia,Rhizedra,Photedes,Oligia,Nonagria,Lenisa,Phragmatiphila,Sedina,Denticucullus,Archanara,Gortyna,Arenostola,Globia,Apamea,Litoligia,Mesoligia,Mesapamea,Luperina,Coenobia,Hydraecia,Helotropha,Amphipoea,Mythimna,Leucania,Noctua,Agrotis) S=89 Q=19 P=12 v1=0.648148 v2=0.683333

(Caradrina,Athetis,Euplexia,Xylena,Lithophane,Calamia,Rhizedra,Photedes,Oligia,Nonagria,Lenisa,Phragmatiphila,Sedina,Denticucullus,Archanara,Gortyna,Arenostola,Globia,Apamea,Litoligia,Mesoligia,Mesapamea,Luperina,Coenobia,Hydraecia,Helotropha,Amphipoea,Mythimna,Leucania,Noctua,Agrotis) S=92 Q=16 P=12 v1=0.703704 v2=0.733333

(Panemeria,Condica,Spodoptera,Pyrrhia,Schinia,Heliothis,Helicoverpa,Elaphria,Cryphia,Caradrina,Athetis,Euplexia,Xylena,Lithophane,Calamia,Rhizedra,Photedes,Oligia,Nonagria,Lenisa,Phragmatiphila,Sedina,Denticucullus,Archanara,Gortyna,Arenostola,Globia,Apamea,Litoligia,Mesoligia,Mesapamea,Luperina,Coenobia,Hydraecia,Helotropha,Amphipoea,Mythimna,Leucania,Noctua,Agrotis,Amphipyra,Acronicta) S=102 Q=24 P=6 v1=0.619048 v2=0.636364

(Condica,Spodoptera,Pyrrhia,Schinia,Heliothis,Helicoverpa,Elaphria,Cryphia,Caradrina,Athetis,Euplexia,Xylena,Lithophane,Calamia,Rhizedra,Photedes,Oligia,Nonagria,Lenisa,Phragmatiphila,Sedina,Denticucullus,Archanara,Gortyna,Arenostola,Globia,Apamea,Litoligia,Mesoligia,Mesapamea,Luperina,Coenobia,Hydraecia,Helotropha,Amphipoea,Mythimna,Leucania,Noctua,Agrotis,Amphipyra,Acronicta) S=102 Q=24 P=6 v1=0.619048 v2=0.636364

(Amphipyra,Acronicta) S=12 Q=18 P=60 v1=-0.2 v2=0.6

(Condica,Spodoptera,Pyrrhia,Schinia,Heliothis,Helicoverpa,Elaphria,Cryphia,Caradrina,Athetis,Euplexia,Xylena,Lithophane,Calamia,Rhizedra,Photedes,Oligia,Nonagria,Lenisa,Phragmatiphila,Sedina,Denticucullus,Archanara,Gortyna,Arenostola,Globia,Apamea,Litoligia,Mesoligia,Mesapamea,Luperina,Coenobia,Hydraecia,Helotropha,Amphipoea,Mythimna,Leucania,Noctua,Agrotis) S=84 Q=40 P=8 v1=0.354839 v2=0.393939

(Trichoplusia,Autographa,Abrostola) S=24 Q=0 P=30 v1=1 v2=1

(Diloba,Deltote) S=6 Q=6 P=12 v1=0 v2=0.5

(Trichoplusia,Autographa) S=12 Q=0 P=42 v1=1 v2=1

(Panemeria,Condica,Spodoptera,Pyrrhia,Schinia,Heliothis,Helicoverpa,Elaphria,Cryphia,Caradrina,Athetis,Euplexia,Xylena,Lithophane,Calamia,Rhizedra,Photedes,Oligia,Nonagria,Lenisa,Phragmatiphila,Sedina,Denticucullus,Archanara,Gortyna,Arenostola,Globia,Apamea,Litoligia,Mesoligia,Mesapamea,Luperina,Coenobia,Hydraecia,Helotropha,Amphipoea,Mythimna,Leucania,Noctua,Agrotis,Amphipyra,Acronicta,Acontia,Panthea,Diloba,Deltote,Cucullia,Trichoplusia,Autographa,Abrostola) S=138 Q=0 P=0 v1=1 v2=1

(Rhizedra,Photedes,Oligia,Nonagria,Lenisa,Phragmatiphila,Sedina,Denticucullus,Archanara,Gortyna,Arenostola,Globia,Apamea,Litoligia,Mesoligia,Mesapamea,Luperina,Coenobia,Hydraecia,Helotropha,Amphipoea) S=18 Q=0 P=12 v1=1 v2=1

(Nonagria,Lenisa,Phragmatiphila,Sedina,Denticucullus,Archanara,Gortyna,Arenostola,Globia,Apamea,Litoligia,Mesoligia,Mesapamea,Luperina,Coenobia,Hydraecia,Helotropha,Amphipoea) S=6 Q=6 P=18 v1=0 v2=0.6

(Gortyna,Arenostola,Globia,Apamea,Litoligia,Mesoligia,Mesapamea,Luperina,Coenobia,Hydraecia,Helotropha,Amphipoea) S=6 Q=6 P=18 v1=0 v2=0.6

(Globia,Apamea,Litoligia,Mesoligia,Mesapamea,Luperina,Coenobia,Hydraecia,Helotropha,Amphipoea) S=0 Q=12 P=18 v1=-1 v2=0.2

(Litoligia,Mesoligia,Mesapamea,Luperina,Coenobia,Hydraecia,Helotropha,AmphipoeaS=0 Q=12 P=0 v1=-1 v2=-1

(Hydraecia,Helotropha,Amphipoea) S=6 Q=6 P=0 v1=0 v2=0

(Hydraecia,Helotropha) S=6 Q=6 P=0 v1=0 v2=0

(Litoligia,Mesoligia,Mesapamea,Luperina,Coenobia) S=0 Q=12 P=0 v1=-1 v2=-1

(Mesoligia,Mesapamea,Luperina,Coenobia) S=6 Q=6 P=0 v1=0 v2=0

(Mesoligia,Mesapamea,Luperina) S=6 Q=6 P=0 v1=0 v2=0

(Mesoligia,Mesapamea) S=12 Q=0 P=0 v1=1 v2=1

(Globia,Apamea) S=12 Q=0 P=18 v1=1 v2=1

(Gortyna,Arenostola) S=0 Q=12 P=0 v1=-1 v2=-1

(Phragmatiphila,Sedina,Denticucullus,Archanara) S=6 Q=6 P=0 v1=0 v2=0

(Phragmatiphila,Sedina,Denticucullus) S=12 Q=0 P=0 v1=1 v2=1

(Cucullia,Trichoplusia,Autographa,Abrostola) S=18 Q=18 P=18 v1=0 v2=0.333333

(Xylena,Lithophane,Calamia) S=6 Q=0 P=30 v1=1 v2=1

(Xylena,Lithophane) S=6 Q=0 P=18 v1=1 v2=1

(Caradrina,Athetis) S=6 Q=0 P=12 v1=1 v2=1

(Elaphria,Cryphia) S=6 Q=0 P=36 v1=1 v2=1

(Sedina,Denticucullus) S=6 Q=6 P=0 v1=0 v2=0

(Nonagria,Lenisa) S=0 Q=12 P=0 v1=-1 v2=-1

(Rhizedra,Photedes,Oligia) S=6 Q=6 P=6 v1=0 v2=0.333333

(Photedes,Oligia) S=0 Q=12 P=6 v1=-1 v2=-0.333333

(Euplexia,Xylena,Lithophane,Calamia,Rhizedra,Photedes,Oligia,Nonagria,Lenisa,Phragmatiphila,Sedina,Denticucullus,Archanara,Gortyna,Arenostola,Globia,Apamea,Litoligia,Mesoligia,Mesapamea,Luperina,Coenobia,Hydraecia,Helotropha,Amphipoea,Mythimna,Leucania,Noctua,Agrotis) S=86 Q=22 P=12 v1=0.592593 v2=0.633333

(Mythimna,Leucania,Noctua,Agrotis) S=62 Q=22 P=30 v1=0.47619 v2=0.614035

(Noctua,Agrotis) S=24 Q=0 P=72 v1=1 v2=1

(Mythimna,Leucania) S=20 Q=4 P=60 v1=0.666667 v2=0.904762

(Euplexia,Xylena,Lithophane,Calamia,Rhizedra,Photedes,Oligia,Nonagria,Lenisa,Phragmatiphila,Sedina,Denticucullus,Archanara,Gortyna,Arenostola,Globia,Apamea,Litoligia,Mesoligia,Mesapamea,Luperina,Coenobia,Hydraecia,Helotropha,Amphipoea) S=12 Q=6 P=30 v1=0.333333 v2=0.75

(Xylena,Lithophane,Calamia,Rhizedra,Photedes,Oligia,Nonagria,Lenisa,Phragmatiphila,Sedina,Denticucullus,Archanara,Gortyna,Arenostola,Globia,Apamea,Litoligia,Mesoligia,Mesapamea,Luperina,Coenobia,Hydraecia,Helotropha,Amphipoea) S=18 Q=0 P=30 v1=1 v2=1

(Pyrrhia,Schinia,Heliothis,Helicoverpa) S=72 Q=0 P=36 v1=1 v2=1

(Schinia,Heliothis,Helicoverpa) S=57 Q=3 P=42 v1=0.9 v2=0.941176

(Heliothis,Helicoverpa) S=54 Q=0 P=42 v1=1 v2=1
