# Supplementary figures and images for: A supertree of Northern European macromoths
Source: PLoS One. 2022 Feb 18;17(2):e0264211. doi: 10.1371/journal.pone.0264211 (PMC8856531; doi:10.1371/journal.pone.0264211)

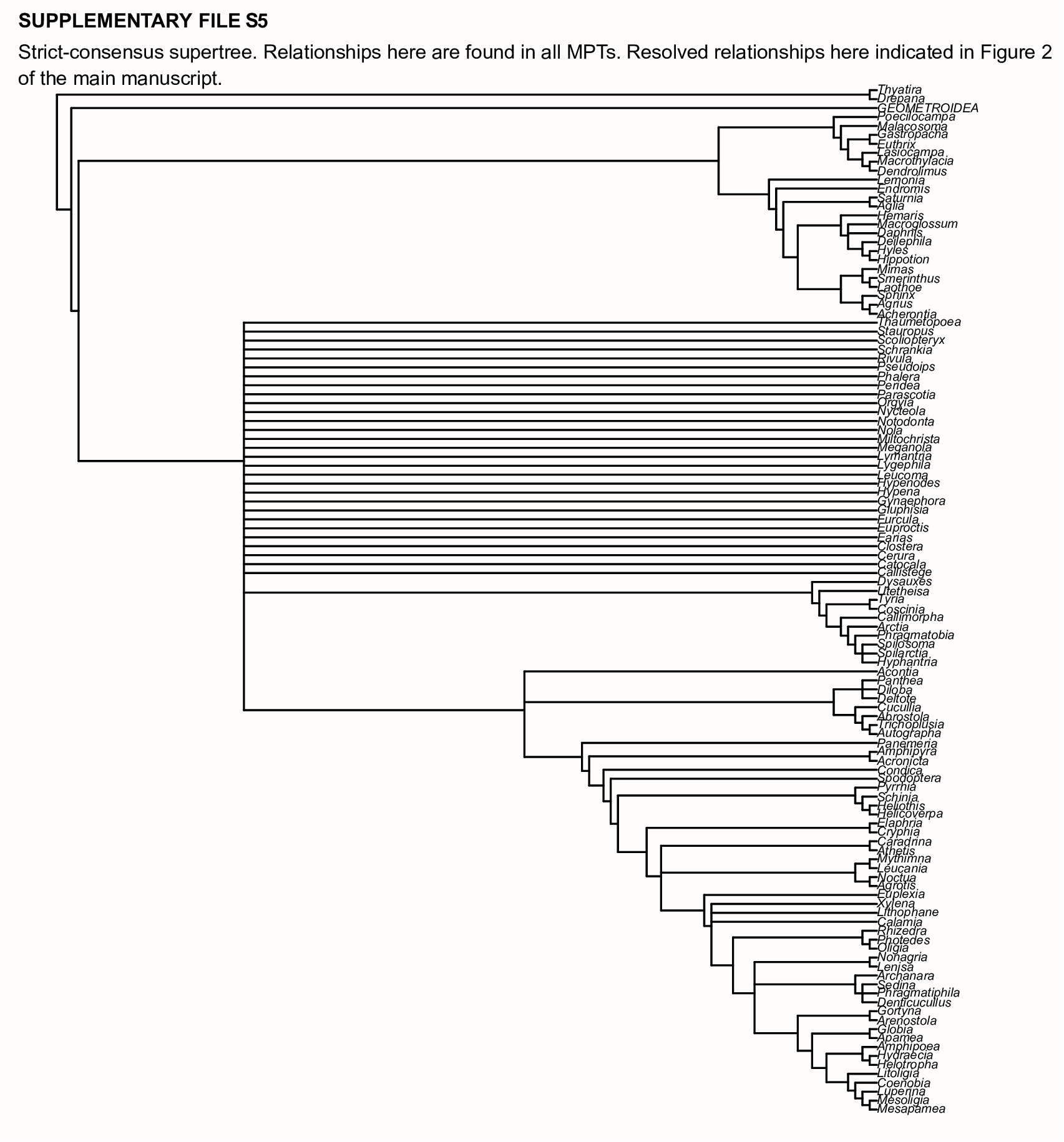

Supplement: S5 File — (TIF) [file pone.0264211.s005.tif]
